# Peripheral Pulsed Radiofrequency for Trigeminal Neuralgia: Early Efficacy with Limited Durability in a Real-World Cohort

**DOI:** 10.3390/jcm15124784

**Published:** 2026-06-19

**Authors:** Gülçin Babaoğlu, Ali Çoştu, Ülkü Sabuncu, Şükriye Dadalı, Nevcihan Şahutoğlu Bal, Şaziye Şahin, Erkan Yavuz Akçaboy

**Affiliations:** Pain Clinic, Ankara Bilkent City Hospital, 06800 Ankara, Türkiye; alicostu@gmail.com (A.Ç.); sabuncuulku@gmail.com (Ü.S.);

**Keywords:** trigeminal neuralgia, pulsed radiofrequency treatment, peripheral nerves, treatment outcome, survival analysis

## Abstract

**Background/Objectives:** Peripheral pulsed radiofrequency (PRF) is a minimally invasive option for trigeminal neuralgia (TN) with a favorable safety profile compared with neurorestorative techniques, but its durability and recurrence patterns remain uncertain. This study evaluated the early effectiveness, durability, recurrence-free survival, and safety of peripheral PRF in refractory classical or idiopathic TN. **Methods:** This retrospective single-center cohort study assessed procedure-level outcomes of peripheral PRF targeting the ophthalmic, maxillary, and mandibular branches. Pain intensity and clinical status were evaluated using the Numeric Rating Scale (NRS) and Barrow Neurological Institute (BNI) pain score. Early effectiveness was defined as clinically meaningful pain relief sustained for at least 1 month, and sustained effectiveness as NRS ≤ 3 at 6 months. Recurrence-free survival was analyzed using Kaplan–Meier methods. **Results:** A total of 68 procedures in 57 patients were analyzed. Early effectiveness at 1 month was achieved in 85.3% of procedures. Median NRS decreased from 9 (IQR 8–9) at baseline to 2 (0–4) at 1 month and 0 (0–2) at 3 and 6 months (*p* < 0.001). In a worst-case analysis, 6-month sustained effectiveness was 72.1%. Recurrence occurred in 61.8% of procedures, with a median recurrence-free survival of 11 months. Among procedures with recurrence, repeat peripheral PRF was performed in 45.2%. Medication requirements decreased in 66.2% of procedures, and no major complications occurred. **Conclusions:** Peripheral PRF provides rapid and meaningful early pain relief in TN, but durability is limited. These findings support peripheral PRF as a safe, repeatable neuromodulatory intervention within a staged treatment strategy rather than a definitive therapy.

## 1. Introduction

Trigeminal neuralgia (TN) is a chronic neuropathic facial pain disorder characterized by recurrent, unilateral, brief episodes of severe stabbing or electric shock-like pain occurring within the distribution of one or more divisions of the trigeminal nerve [1,2]. Because it is triggered by otherwise innocuous daily activities such as speaking, eating, drinking, and light touch, it markedly impairs daily functioning and substantially reduces quality of life by contributing to social isolation, anorexia, and sleep disturbance [3,4].

Pharmacotherapy with carbamazepine and oxcarbazepine remains the gold standard for first-line management; however, long-term treatment is frequently limited by inadequate response, dose-limiting adverse effects, or both [5,6,7]. Accordingly, many patients eventually require interventional treatment.

Interventional treatment options encompass a broad spectrum, ranging from peripheral nerve branch procedures to percutaneous ablative interventions targeting the Gasserian ganglion—such as radiofrequency thermocoagulation (RFT), balloon compression, and glycerol rhizolysis—and microvascular decompression (MVD) [8,9,10]. Although conventional Gasserian ganglion RFT and other neurodestructive techniques demonstrate high success rates, they are associated with significant risks, including permanent facial numbness (hypoesthesia), masseter muscle weakness, and anesthesia dolorosa [11]. In cases involving the ophthalmic division, high-temperature Gasserian ganglion RFT may result in serious ocular complications, including loss of the corneal reflex, keratitis, and even blindness [12]. To avoid these neurodestructive complications and provide a safer alternative for patients who are not suitable candidates for invasive surgical procedures, pulsed radiofrequency (PRF) targeting the peripheral branches of the trigeminal nerve (ophthalmic, maxillary, and mandibular nerves) has emerged as a contemporary treatment option [13,14,15].

The application of PRF to peripheral nerve branches—particularly when performed under ultrasound guidance—allows real-time visualization of soft tissues and vascular structures, thereby potentially reducing the risk of injury to adjacent tissues compared with landmark-based techniques [16,17,18]. The broader PRF literature also demonstrates considerable heterogeneity across studies in terms of technical parameters and follow-up definitions [19]. Nevertheless, the durability of its efficacy remains a matter of debate.

Cohort studies of peripheral terminal branch PRF report meaningful short-term analgesia [13,20]; however, comparative data suggest that outcome differences may become more pronounced over time when peripheral PRF is contrasted with more destructive Gasserian approaches [14,15]. In this context, evaluating peripheral PRF solely through short-term pain reduction may underestimate clinically relevant issues such as recurrence, retreatment need, and loss to follow-up. Therefore, this study aimed to evaluate the outcomes of peripheral PRF with explicit consideration of informative missing data, recurrence timing, and procedure-level retreatment patterns.

## 2. Materials and Methods

This study was designed as a single-center, retrospective observational cohort. The study protocol was approved by the local ethics committee, and all procedures were conducted in accordance with the Declaration of Helsinki. Due to the retrospective nature of the study, informed consent was not obtained from the patients. To ensure confidentiality, all patient data were anonymized before data extraction and analysis, preventing the identification of individual patients. Medical records of patients who underwent pulsed radiofrequency treatment targeting the ophthalmic, maxillary, and/or mandibular nerves for trigeminal neuralgia at a tertiary pain management clinic between January 2022 and June 2025 were retrospectively reviewed. The reporting of this study follows the Strengthening the Reporting of Observational Studies in Epidemiology (STROBE) guidelines (Supplemental Table S1).

### 2.1. Patient Selection

In this retrospective cohort study, we included patients aged ≥ 18 years with classical or idiopathic trigeminal neuralgia who underwent PRF treatment targeting the peripheral branches of the trigeminal nerve (ophthalmic, maxillary, and/or mandibular). The diagnosis of the trigeminal neuralgia was established according to the International Classification of Headache Disorders (ICHD-3) criteria [2]. Prior to PRF treatment, all patients had undergone appropriate clinical and radiological evaluation, including magnetic resonance imaging (MRI), to exclude secondary causes of trigeminal neuralgia and other conditions associated with trigeminal neuropathy. Patients were considered candidates for PRF if they had failed at least one adequate trial of carbamazepine or oxcarbazepine, defined as insufficient pain control at the maximum tolerated dose or inability to tolerate these agents because of adverse effects. In addition, all included patients had received at least one analgesic medication before undergoing PRF treatment. Therefore, conservative management consisted primarily of pharmacological therapy, including carbamazepine or oxcarbazepine and adjunct analgesic medications.

**Figure 1:**
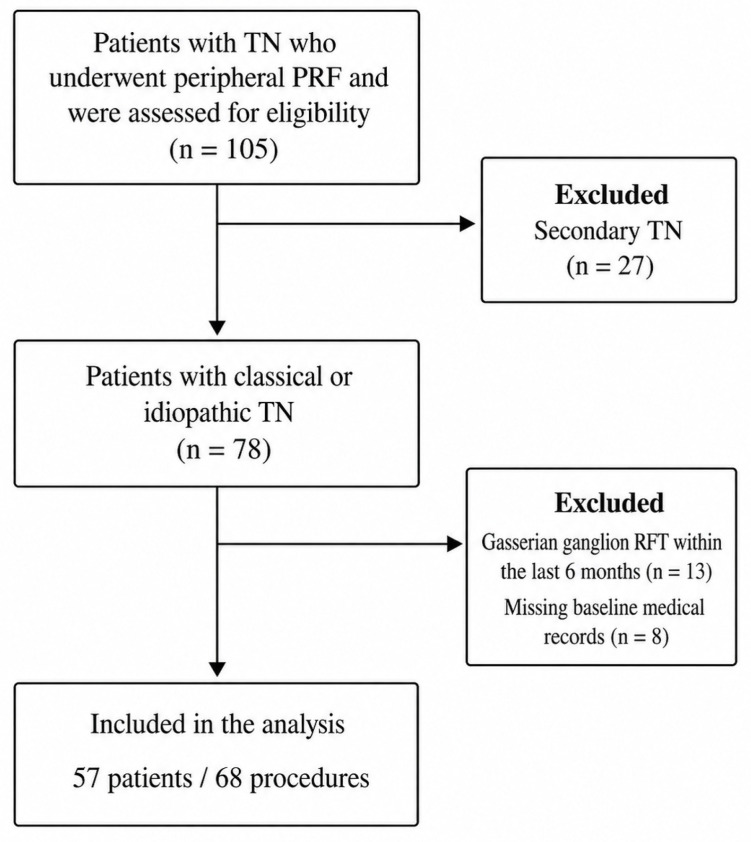
Flowchart of study. The final analysis included 57 patients and 68 procedures; 9 patients underwent the procedure twice and 1 patient three times. Abbreviations: TN, trigeminal neuralgia; PRF, pulsed radiofrequency; RFT, radiofrequency thermocoagulation.

Patients with secondary trigeminal neuralgia (e.g., multiple sclerosis or cerebellopontine angle tumor), as well as patients with painful trigeminal neuropathy, those who had undergone Gasserian ganglion RFT or MVD within 6 months before peripheral PRF, and those with missing baseline data were excluded. The unit of analysis was the procedure rather than the patient. The study was conducted within a tertiary pain clinic workflow and analyzed as real-world practice data. The patient selection and exclusion process is summarized in Figure 1. A positive response to a pre-procedural diagnostic peripheral local anesthetic block (at least 50% pain reduction) was required.

Patients with secondary trigeminal neuralgia (e.g., multiple sclerosis or cerebellopontine angle tumor), those who had undergone Gasserian ganglion RFT or MVD within 6 months before peripheral PRF, and those with missing baseline data were excluded. The unit of analysis was the procedure rather than the patient. The study was conducted within a tertiary pain clinic workflow and analyzed as real-world practice data.

### 2.2. Procedures

All procedures were performed in an operating room setting under continuous monitoring, with patients in the supine position. PRF was applied to the symptomatic peripheral branch or branches of the trigeminal nerve according to the anatomical distribution of the patient’s pain. When multiple branches were involved, all affected branches were treated in the same session. A 22G radiofrequency cannula with a 5 mm active tip (SMK™, 22 GA × 5 cm, Abbott Medical, Plymouth, MN, USA) was advanced toward the target nerve.

For the ophthalmic division, patients were positioned with the head in a neutral position, and the cannula was advanced from lateral to medial toward the inferolateral aspect of the supraorbital notch. For the maxillary division, slight neck extension and contralateral head rotation were applied; under ultrasound guidance, the maxillary artery, lateral pterygoid plate, and adjacent anatomical structures were identified, and the cannula was advanced using an out-of-plane technique to target the maxillary nerve. For the mandibular division, with the mouth open, a transducer was placed below the zygomatic arch to identify the lateral pterygoid plate, and the cannula was advanced using an out-of-plane approach toward its posterior border.

Target confirmation was performed for each treated branch prior to PRF using sensory stimulation at 50 Hz. Correct needle placement was confirmed by evoking concordant paresthesia within the painful distribution at a stimulation threshold of 0.3–0.7 V. For mandibular procedures, additional motor stimulation at 2 Hz (1–2 V) was used to evoke masseter muscle contraction. PRF was then delivered using standardized parameters (42 °C, 2 Hz, 20 ms pulse width) as a single continuous 360 s (6 min) cycle per branch. In cases involving multiple trigeminal branches, each affected branch was treated separately and received a full 360 s PRF application, with target confirmation repeated before treatment of each branch.

All patients were routinely monitored in an observation unit for at least 60 min after the procedure and were discharged after completion of general and neurological assessments. All procedures were performed by physicians experienced in interventional pain management using a standardized procedural protocol.

### 2.3. Data Collection

All clinical data were retrospectively obtained from the hospital electronic medical record system. The dataset comprised 68 procedures performed in 57 unique patients. Ten patients underwent more than one intervention, contributing 21 procedures in total; specifically, 9 patients underwent two procedures each, while 1 patient underwent three procedures. As the clinical objective was to evaluate procedure-level effectiveness within a sequential care framework, repeated procedures from the same patient were retained as independent observations in the analysis.

Demographic and clinical variables included age, sex, disease duration, comorbidities, pain pattern (paroxysmal, paroxysmal with constant, or constant), pain laterality, trigeminal nerve distribution, magnetic resonance imaging findings (presence or absence of neurovascular conflict), prior interventional treatment history (Gasserian RFT, MVD, peripheral PRF), and current pharmacologic therapy before the procedure. Baseline pain intensity and clinical status were assessed using the Numeric Rating Scale (NRS) and Barrow Neurological Institute (BNI) scores.

For missing data management, procedures lacking baseline data were excluded, and follow-up data were analyzed according to available-case principles. No data imputation was performed for other missing or unavailable variables, and all analyses were conducted using available data only.

### 2.4. Outcome Measures

Pain intensity was assessed using the NRS, a subjective measure ranging from 0 (no pain) to 10 (worst pain imaginable). Clinical status was additionally evaluated using the BNI pain score, an ordinal classification that incorporates both pain severity and the need for pharmacologic treatment and is widely used in the assessment of trigeminal neuralgia outcomes (Table 1) [21].

Treatment effectiveness was evaluated at two levels. Early effectiveness was defined as clinically meaningful pain relief sustained for at least 1-month after the procedure. Procedures requiring escalation to an advanced intervention within the first month were classified as ineffective. Sustained effectiveness was operationally defined as clinically meaningful improvement at the 6-month assessment, corresponding to maintenance of a mild pain or pain-free state (NRS ≤ 3) at the 6-month assessment. Recurrence was defined as the reappearance of pain after an initial successful response, manifested by the clinical need for second-line interventional treatment or an adjustment in the therapeutic regimen. To ensure analytical consistency, any procedure that failed to maintain an NRS ≤ 3 at the 6-month assessment, in the absence of an earlier re-intervention, was operationally classified as a recurrence event.

Because missing 6-month data were clinically informative (all missing cases had early recurrence and underwent re-intervention), two analytical approaches were reported:

- Available-case analysis: Denominator = Procedures with available 6-month NRS data.
- Worst-case procedure-level analysis: Denominator = All procedures, with missing 6-month outcomes considered treatment failures.

Secondary outcome measures included recurrence and time to recurrence, post-procedure medication status (discontinued, decreased, unchanged, or increased), second-line interventions, and complications. For time-to-event analyses, time zero was defined as the procedure date. Time to recurrence was defined as the interval from the procedure to the first recurrence event (pain relapse or re-intervention).

### 2.5. Statistical Analysis

Continuous variables are presented as mean ± SD when helpful for baseline description, and as median (IQR) for non-normally distributed outcomes. Normality was assessed using the Shapiro–Wilk test. Categorical variables are reported as counts and percentages.

Within-subject temporal changes were tested using Friedman tests (complete cases across all four time points). Pairwise differences were assessed using Wilcoxon signed-rank tests with Bonferroni correction for six comparisons. Exploratory comparisons between effective and non-effective procedures used Mann–Whitney U tests for continuous variables and chi-square or Fisher exact tests for categorical variables. Recurrence-free survival was estimated using Kaplan–Meier methods with censoring at the last known follow-up. As a sensitivity analysis, effectiveness and recurrence outcomes were re-evaluated using only the first procedure per patient to assess the potential impact of repeated procedures on the primary findings. Prespecified subgroup recurrence-free survival comparisons were performed using log-rank tests for idiopathic and classical TN, prior interventional treatment status (treatment-naive vs. previously treated) and pain pattern (paroxysmal vs. mixed/constant). A two-sided *p* value < 0.05 was considered statistically significant.

## 3. Results

### 3.1. Baseline Clinical Characteristics

A total of 68 peripheral PRF procedures were performed in 57 patients. At the patient level (*n* = 57), 34 patients (59.6%) were female; at the procedure level (*n* = 68), 37 procedures (54.4%; 95% CI 42.7–65.7%) were performed in female patients. The median age was 60 years (IQR 49–72; range 24–83). Median disease duration was 60 months (IQR 33–120; range 6–360). At least one comorbid condition was present in 46 procedures (67.6%; 95% CI 55.8–77.6%). Neurovascular conflict on MRI was identified in 18 procedures (26.5%; 95% CI 17.4–38.0%).

Pain was paroxysmal in 48 procedures (70.6%; 95% CI 58.9–80.1%), mixed constant-paroxysmal in 19 (27.9%), and purely constant in 1 (1.5%). The right trigeminal nerve was affected in 45 procedures (66.2%), the left in 22 (32.4%), and both sides in 1. The maxillary branch was the most commonly involved distribution (35.3%), followed by the mandibular branch (22.1%) and combined maxillary and mandibular involvement (22.1%). Forty-three procedures (63.2%) were performed in patients without prior interventional treatment; 25 (36.8%; 95% CI 26.3–48.6%) had undergone at least one previous procedure, most commonly Gasserian ganglion RFT (*n* = 17) or peripheral PRF (*n* = 16).

Carbamazepine was used in 61 procedures (89.7%; 95% CI 80.2–94.9%) at a median dose of 800 mg/day (IQR 600–1200). Gabapentin, pregabalin, duloxetine, and baclofen were used in 35.3%, 22.1%, 8.8%, and 8.8% of procedures, respectively. Detailed baseline data are shown in Table 2.

### 3.2. Treatment Effectiveness

The initial responder rate at 1 month was 85.3% (*n* = 58/68) of procedures. In the intent-to-treat analysis (*n* = 68), sustained effectiveness (NRS ≤ 3) was 72.1% (95% CI 60.4–81.3%) at 6 months (Table 3). Of the 19 failures (27.9%), 14 required re-intervention before the 6-month assessment and were classified as failures due to informative censoring, while 5 failed to meet the response threshold despite available follow-up data. Detailed effectiveness rates are summarized in Table 3.

Baseline NRS was the only variable significantly associated with the clinical outcome: effective procedures had a lower median baseline NRS (8.0 [IQR 8.0–9.0]) compared to failures (9.0 [IQR 8.5–9.0]; *p* = 0.024). Age, sex, disease duration, MRI findings, pain pattern, branch distribution, and prior treatment history did not differ significantly between groups (all *p* > 0.05).

### 3.3. NRS Scores over Time

Pain intensity decreased significantly from a median NRS of 9 (IQR 8–9) at baseline to 2 (IQR 0–4) at 1 month, reaching 0 (IQR 0–2) at both 3 and 6 months (*p* < 0.001). Notably, while the median NRS was 0 at the 6-month mark, complete pain-free status (NRS = 0) was maintained in 33.8% of the total cohort. This indicates that the median value primarily reflects the results of sustained responders, whereas the overall effectiveness is more accurately captured by the worst-case analysis presented in Table 3.

### 3.4. BNI Distribution over Time

**Figure 2:**
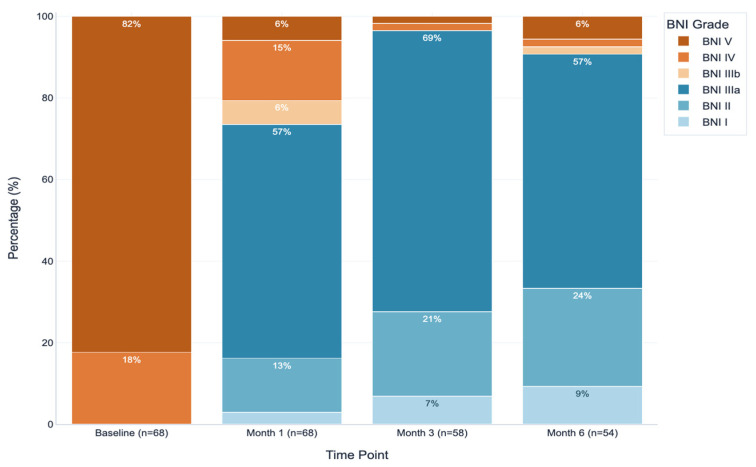
Barrow Neurological Institute (BNI) grade distribution at baseline and at 1, 3, and 6 months. The proportion of patients with severe pain-related disability decreased after treatment, while favorable BNI grades increased during follow-up. Stacked bars show the percentage distribution of BNI grades at each time point.

At baseline, all procedures were classified as BNI grade IV (17.6%) or V (82.4%). By month 1, 57.4% reached BNI IIIa and 16.2% achieved BNI I or II. By month 3, 69.0% were classified as BNI IIIa and 27.6% as BNI I or II. At month 6, 9.3% achieved complete pain relief (BNI I), and 24.1% were classified as BNI II. BNI scores improved significantly at all follow-up time points (all *p* < 0.001) (Figure 2).

### 3.5. Recurrence and Kaplan–Meier Survival Analysis

**Figure 3:**
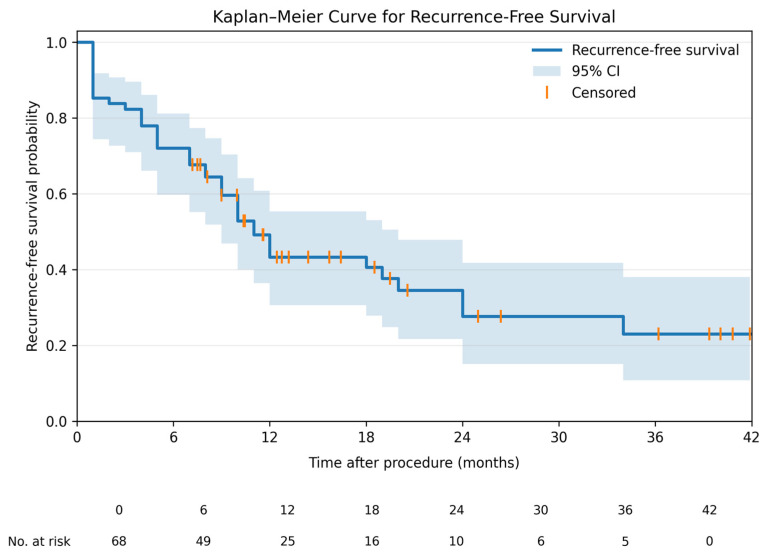
Kaplan–Meier recurrence-free survival after treatment. The Kaplan–Meier curve shows recurrence-free survival over follow-up, with the shaded area representing the 95% confidence interval. Recurrence-free survival was 72.1% at 6 months and 44.3% at 12 months; median recurrence-free survival was 11 months. Numbers below the x-axis indicate patients at risk.

Pain recurrence occurred in 42 procedures (61.8%; 95% CI 49.9–72.4%). Median time to recurrence was 7 months (IQR 2–11; range 1–34). Kaplan–Meier analysis used each procedure’s actual follow-up duration for censoring; recurrence-free procedures were observed for 8.1 to 42.8 months and were not artificially capped at 6 months. This approach preserves all available information from patients with longer follow-up. Median recurrence-free survival was 11 months (Figure 3). Of the 42 procedures with recurrence, 19 underwent repeat peripheral PRF and 15 underwent Gasserian ganglion RFT as second-line treatment.

As a sensitivity analysis, outcomes were also evaluated using only the first procedure per patient (*n* = 57). Six-month effectiveness was 73.7%, recurrence rate was 64.9%, and median recurrence-free survival was 11 months, closely matching the estimates obtained in the primary procedure-level analysis.

Six month effectiveness was comparable between classical and idiopathic TN (classical 66.7% vs. idiopathic 74.0%; Fisher’s exact *p* = 0.555). Also, recurrence-free survival did not differ significantly between classical and idiopathic TN subgroups (median 11 vs. 12 months; 6-month survival 66.7% vs. 74.0%; log-rank *p* = 0.569), between treatment-naive and previously treated procedures (log-rank *p* = 0.367), or between paroxysmal and mixed/constant pain patterns (log-rank *p* = 0.904).

### 3.6. Medication Status and Safety

Medications were discontinued in 13 procedures (19.1%), dose-reduced in 32 (47.1%), unchanged in 17 (25.0%), and increased in 6 (8.8%). Overall, 45 procedures (66.2%; 95% CI 54.3–76.3%) resulted in medication discontinuation or dose reduction.

One complication was recorded: transient dizziness (1.5%; 95% CI 0.3–8.0%). No facial hypoesthesia, masseter weakness, diminished corneal reflex, hematoma, or infection were observed.

## 4. Discussion

In this real-world cohort, peripheral PRF for trigeminal neuralgia provided rapid and significant early analgesia, accompanied by meaningful functional improvement. However, recurrence occurred in approximately two-thirds of the procedures, with a median recurrence-free survival of 11 months. Importantly, recurrence did not necessarily imply clinical failure; a substantial proportion of patients were successfully managed with repeat peripheral PRF. These findings indicate that although peripheral PRF offers a potent short-term effect, its limited durability supports its use as part of a repeatable and staged treatment strategy.

The apparent discordance between the high initial success rate (85.3%) and the subsequent recurrence rate (61.8%) highlights the temporal nature of peripheral PRF efficacy. Although the 6-month effectiveness remained robust at 72.1% under a comprehensive worst-case analysis, the high cumulative recurrence underscores that the procedure functions as a potent but time-limited neuromodulatory intervention. This pattern indicates that while many patients maintain a significant reduction in pain at 6 months, a substantial proportion eventually require re-intervention during longer-term follow-up. Consequently, the success of peripheral PRF should be defined by its ability to provide repeatable periods of analgesia rather than permanent remission.

These findings are consistent with previous studies demonstrating that peripheral PRF provides effective early-term pain control with a safer profile than neurodestructive techniques [13,14]. Ultrasound-guided PRF applied to peripheral branches has been reported to achieve significant analgesia without major complications [20]. Similarly, Can et al. reported that maxillary and mandibular nerve PRF was effective over a 3-month follow-up period and identified advanced age, higher preprocedural NRS scores, and non-idiopathic etiology as independent predictors of poor response [13]. In our analysis, however, only baseline pain severity showed a limited association with treatment effectiveness, whereas no other clinical predictor was identified.

The balance between peripheral PRF and interventions targeting the Gasserian ganglion remains unclear. In a randomized controlled trial, both Gasserian RFT and maxillary/mandibular PRF achieved effective pain control at 6 months, with a higher—but not statistically significant—success rate in the RFT group [14]. In contrast, a retrospective cohort found that although both techniques were effective early, the effect of peripheral PRF declined at 3 and 6 months, whereas Gasserian RFT showed more sustained results [15]. Taken together, these findings suggest that peripheral PRF provides good early pain relief, while Gasserian-level interventions may offer more durable outcomes in selected patients.

From a clinical perspective, the main advantage of peripheral PRF is its favorable risk–benefit ratio. Although MVD provides the highest long-term satisfaction and the lowest recurrence rates, it is the most invasive option [22] and is associated with severe complications, including a mortality rate of approximately 0.1–0.3% [23,24]. For patients who are ineligible for craniotomy or who decline surgery, percutaneous ganglion lesioning or Gamma Knife stereotactic radiosurgery represent effective alternatives [6,25]. Gasserian ganglion RFT can achieve prolonged pain relief through thermocoagulation of nociceptive fibers, but is associated with complications such as corneal reflex loss, keratitis, facial hypoesthesia, and masticatory weakness [26,27]. Conversely, PRF achieves analgesia without inducing neurodestruction by maintaining tissue temperature below the ablation threshold; because of this non-destructive property, it stands out as a safe alternative [28].

Durability remains the primary limitation of peripheral PRF. In this cohort, a recurrence rate of 61.8%, a median time to recurrence of 7 months, and a median recurrence-free survival of 11 months indicate limited long-term efficacy. The literature similarly reports that the duration of PRF efficacy varies depending on the application technique and the targeted anatomical level. In studies focusing on peripheral branches, pain control was achieved in 54.2% of patients for at least 3 months [13], while another series reported at least 50% reduction in NRS scores in 63.6% of cases [14]. However, long-term data show that recurrence-free survival declines over time [29]. In contrast, higher and more persistent response rates have been reported at the Gasserian ganglion level [30]. These findings suggest that peripheral PRF provides a more limited but safer analgesic profile.

In most cases, recurrence does not indicate clinical failure. The fact that 45.2% of recurrences were successfully managed with repeat peripheral PRF, and that prior interventional treatment did not significantly affect recurrence-free survival, supports the repeatability of this approach within sequential treatment pathways. Consistent with the literature, despite its limited durability, PRF remains a practical neuromodulatory option because of its low complication profile and can be safely reapplied when needed [31].

In this context, peripheral PRF should be evaluated as a component of an individualized and staged treatment strategy rather than a one-time definitive intervention. In cases where adequate early-term analgesia is not achieved, the integration of techniques targeting more proximal anatomical levels into the treatment algorithm may represent a rational approach.

Key methodological strengths of this study include the procedure-level analysis approach, conservative handling of informative missing data, and the use of time-to-event analyses. Specifically, by applying a worst-case scenario analysis to evaluate 6-month effectiveness, we classified all cases lost to follow-up due to early recurrence as treatment failures. This conservative approach provides a more transparent dataset than available-case analysis, which can artificially inflate success rates. Consequently, the reported 72.1% success rate represents a clinically realistic outcome that prioritizes clinical integrity and accurately reflects the performance of peripheral PRF in a refractory TN population.

However, several limitations should be acknowledged, including the retrospective single-center design, the absence of a control group, non-random missingness in 6-month follow-up data, small statistical power for subgroup analyses, and the limited availability of patient-reported outcomes. In addition, patient diversity was limited. The cohort was derived from a single tertiary pain clinic and consisted predominantly of patients with idiopathic trigeminal neuralgia, with a relatively narrow baseline high pain severity distribution. These characteristics may limit the generalizability of the findings to broader trigeminal neuralgia populations, particularly patients with classical TN or lower baseline pain severity. A further methodological consideration is that repeat procedures in the same patient were analyzed as independent observations. Although this approach may not fully account for within-patient correlation and may slightly underestimate standard errors, it was chosen to reflect real-world sequential care, in which each recurrence represents a distinct clinical decision point. This procedure-level framework allows peripheral PRF to be evaluated pragmatically as a repeatable, staged intervention rather than as a one-time definitive treatment.

## 5. Conclusions

Peripheral PRF provides rapid and clinically meaningful pain relief in patients with refractory trigeminal neuralgia, with a low complication profile and a substantial reduction in medication burden. However, its durability appears limited, and recurrence should be anticipated during long-term follow-up. These findings suggest that peripheral PRF should not be regarded as a stand-alone definitive treatment, but rather as a safe, repeatable neuromodulatory intervention within a strategic and staged treatment approach.

## Figures and Tables

**Table 1:** BNI Pain Intensity Score.

Scores	Definition
BNI I	No pain, no medication
BNI II	Occasional pain, no medication required
BNI IIIa	No pain, continued medication
BNI IIIb	Some pain, adequately controlled with medication
BNI IV	Some pain, not adequately controlled with medication
BNI V	Severe pain/no pain relief

Abbreviation: BNI = Barrow Neurological Institute.

**Table 2:** Baseline Characteristics (*n* = 68 procedures, 57 patients).

Variable	Value
Age, years (median [IQR])	60 [49–72]
Female sex, *n* (%)	37 (54.4%)
Disease duration, months (median [IQR])	60 [33–120]
At least one comorbidity, *n* (%)	46 (67.6%)
MRI neurovascular conflict, *n* (%)	18 (26.5%)
Paroxysmal pain pattern, *n* (%)	48 (70.6%)
Affected side, right	45 (66.2%)
Pain distribution	
Maxillary branch	24 (35.3%)
Mandibular branch	15 (22.1%)
Maxillary + mandibular branches	15 (22.1%)
Prior interventional treatment, *n* (%)	25 (36.8%)
Carbamazepine use, *n* (%)	61 (89.7%)
Carbamazepine dose, mg/day (median [IQR])	800 [600–1200]
Baseline NRS (median [IQR])	9.0 [8.0–9.0]
Baseline BNI Grade V— *n* (%)	56 (82.4%)
Baseline BNI Grade IV— *n* (%)	12 (17.6%)

Abbreviations: BNI = Barrow Neurological Institute, IQR = interquartile range; MRI = magnetic resonance imaging; NRS = Numeric Rating Scale.

**Table 3:** Clinical Effectiveness Rates Over Time (*n* = 68).

Time Point	Clinical Success (NRS ≤ 3)	Complete Pain Relief (NRS = 0)
1 Month	85,3% (*n* = 58)	22.1% (*n* = 15)
3 Months	80.9% (*n* = 55)	60.3% (*n* = 41)
6 Months	72.1% (*n* = 49)	33.8% (*n* = 23)

Note: Percentages are calculated using a worst-case analysis. Abbreviations: NRS = Numeric Rating Scale.

## Data Availability

The raw data supporting the conclusions of this article will be made available by the corresponding author, G.B., upon reasonable request.

## Supplementary Materials

The following supporting information can be downloaded at: https://www.mdpi.com/article/10.3390/jcm15124784/s1, Table S1: STROBE Checklist for Consort Studies.

## Abbreviations

The following abbreviations are used in this manuscript:

BNI	Barrow Neurological Institute
CI	Confidence interval
ICHD-3	International Classification of Headache Disorders, 3rd edition
IQR	Interquartile range
MRI	Magnetic resonance imaging
MVD	Microvascular decompression
NRS	Numeric Rating Scale
PRF	Pulsed radiofrequency
RFS	Recurrence-free survival
RFT	Radiofrequency thermocoagulation
TN	Trigeminal neuralgia
