# Supplementary material for: Peripheral Pulsed Radiofrequency for Trigeminal Neuralgia: Early Efficacy with Limited Durability in a Real-World Cohort
Source: J Clin Med. 2026 Jun 19;15(12):4784. doi: 10.3390/jcm15124784 (PMC13300827; doi:10.3390/jcm15124784)
Supplement: Supplementary file 1 [file jcm-15-04784-s001.zip › jcm-4317978-supplementary.pdf]

Supplementary Table S1: STROBE Checklist for Cohort Studies

Manuscript: Peripheral Pulsed Radiofrequency for Trigeminal Neuralgia: Early Efficacy with Limited Durability in a Real-World Cohort **Journal:** Journal of Clinical Medicine **Manuscript ID:** JCM-4317978

Section / Topic	Item No.	Checklist Item	Reported on Page / Line
**TITLE AND ABSTRACT**			
Title and abstract	1a	Indicate the study's design with a commonly used term in the title or the abstract	Title (p.1): "…Real-World Cohort"; Abstract Methods (p.1, line 18): "retrospective single-center cohort study"
	1b	Provide in the abstract an informative and balanced summary of what was done and what was found	Abstract, p.1, lines 13–33
**INTRODUCTION**			
Background / rationale	2	Explain the scientific background and rationale for the investigation being reported	Introduction, p.1–2, lines 37–74
Objectives	3	State specific objectives, including any prespecified hypotheses	Introduction, p.2, lines 72–74: "…this study aimed to evaluate the outcomes of peripheral PRF with explicit consideration of informative missing data, recurrence timing, and procedure-level retreatment patterns."
**METHODS**			
Study design	4	Present key elements of study design early in the paper	Materials and Methods, p.3, lines 77–78: "single-center, retrospective observational cohort"
Setting	5	Describe the setting, locations, and relevant dates, including periods of recruitment, exposure, follow-up, and data collection	Materials and Methods, p.3, lines 77–87: tertiary pain clinic, Ankara Bilkent City Hospital; January 2022 – June 2025
Participants	6a	Cohort study — Give the eligibility criteria, and the sources and methods of selection of participants	Patient Selection, p.3, lines 89–106; Figure 1 (flowchart), p.4
	6b	Cohort study — For matched studies, give matching criteria and number of exposed and unexposed	Not applicable (no matching)
Variables	7	Clearly define all outcomes, exposures, predictors, potential confounders, and effect modifiers. Give diagnostic criteria, if applicable	Data collection, p.5, lines 153–170; Outcome Measures, lines 172–176

Data sources / measurement	8	For each variable of interest, give sources of data and details of methods of assessment (measurement). Describe comparability of assessment methods if there is more than one group	Data collection, p.5, lines 153–159: hospital electronic medical records; NRS (0–10) and BNI score (Table 1)
Bias	9	Describe any efforts to address potential sources of bias	Discussion / Limitations, p.11, lines 394–402: retrospective design, selection bias, informative censoring acknowledged; worst-case analysis applied to address informative missing data
Study size	10	Explain how the study size was arrived at	Data collection p.5, lines 153–159: all consecutive eligible procedures within the study period were included; no formal sample size calculation (retrospective, all-available-cases design)
Quantitative variables	11	Explain how quantitative variables were handled in the analyses. If applicable, describe which groupings were chosen and why	Statistical Analysis, p.6, lines 201-203: Shapiro-Wilk normality testing; non-normally distributed variables reported as median [IQR]; NRS and BNI treated as continuous/ordinal
Statistical methods	12a	Describe all statistical methods, including those used to control for confounding	Statistical Analysis, p.6, lines 204–210: Friedman test, Wilcoxon signed-rank with Bonferroni correction, Mann-Whitney U, Fisher's exact, Kaplan-Meier with log-rank
	12b	Describe any methods used to examine subgroups and interactions	Statistical Analysis, p.6, lines 210–213: prespecified subgroup log-rank comparisons for prior treatment status, pain pattern, and TN subtype (classical vs. idiopathic)
	12c	Explain how missing data were addressed	Outcome Measures, p.5, lines 190–199: two analytical approaches — available-case and worst-case procedure-level analysis; missing 6-month data classified as treatment failures due to informative censoring
	12d	Cohort study — If applicable, explain how loss to follow-up was addressed	Outcome Measures, p.5, lines 179–184 procedures without 6-month data had all experienced early recurrence and were classified as failures (informative censoring, not random loss)
	12e	Describe any sensitivity analyses	Statistical Analysis, p.6 lines 210-212: patient-level sensitivity analysis restricting to first procedure per patient
**RESULTS**			
Participants	13a	Report numbers of individuals at each stage of study — e.g., numbers potentially eligible, examined, confirmed eligible, included in the study, completing follow-up, and analysed	Figure 1 (flowchart), p.4: 105 assessed → 27 excluded (secondary TN) → 78 eligible → 21 excluded (Gasserian RFT within 6 mo, n=13; missing baseline, n=8) → 57 patients / 68 procedures analysed
	13b	Give reasons for non-participation at each stage	Figure 1, p.4: exclusion reasons specified (secondary TN, recent Gasserian RFT, missing baseline data)
	13c	Consider use of a flow diagram	Figure 1, p.4: CONSORT-style flowchart provided

Descriptive data	14a	Give characteristics of study participants (e.g., demographic, clinical, social) and information on exposures and potential confounders	Table 2 (Baseline Characteristics), p.6; lines 219–238
	14b	Indicate number of participants with missing data for each variable of interest	Outcome measures, p.5, lines 190–111;<br>Treatment Effectiveness, p.7-8, lines 243–248
	14c	Cohort study — Summarise follow-up time (e.g., average and total amount)	Recurrence and Kaplan-Meier Survival Analysis, p.9: lines 276-293<br>median follow-up for the full cohort was 19 months (IQR 14–32; range 8–43; mean 22.5 ± 11.0). Among procedures without recurrence (n = 26), median follow-up was 15 months (IQR 11–25). KM analysis censored each procedure at its actual follow-up date.
Outcome data	15	Cohort study — Report numbers of outcome events or summary measures over time	Table 3 (Effectiveness Rates), p.8; Figure 3 (KM estimates), p.10; Treatment effectiveness, NRS scores over time, BNI distribution over time, recurrence and Kaplan-meier survival analysis p.7–9, lines 243-293
Main results	16a	Give unadjusted estimates and, if applicable, confounder-adjusted estimates and their precision (e.g., 95% CI)	Treatment effectiveness, p.7, lines 243-248: effectiveness 72.1% (95% CI 60.4–81.3%); Recurrence and Kaplan-Meier Survival Analysis, p.9, lines 276,283: KM estimates with 95% CI ; Medication Status and Safety, p.10, 300-305: medication 66.2% (95% CI 54.3–76.3%)
	16b	Report category boundaries when continuous variables were categorized	Not applicable; NRS and BNI reported as continuous/ordinal distributions
	16c	If relevant, consider translating estimates of relative risk into absolute risk for a meaningful time period	KM absolute recurrence-free survival probabilities reported at 1, 3, 6, 9, 12, 18, 24 months (Figure 3, p.10)
Other analyses	17	Report other analyses done — e.g., analyses of subgroups and interactions, and sensitivity analyses	Recurrence and Kaplan-Meier Survival Analysis, p.9, lines 288–293
**DISCUSSION**			
Key results	18	Summarise key results with reference to study objectives	Discussion, p.10, lines 307–314: key findings summarised in opening paragraph
Limitations	19	Discuss limitations of the study, taking into account sources of potential bias or imprecision. Discuss both direction and magnitude of any potential bias	Discussion, p.12, lines 388–402: retrospective design, single-center selection bias, no control group, informative censoring, within-patient correlation not modeled, limited patient diversity, narrow NRS distribution
Interpretation	20	Give a cautious overall interpretation of results considering objectives, limitations, multiplicity of	Discussion, p.10–12, lines 307–387: findings interpreted in context of published peripheral PRF and Gasserian PRF literature; limitations explicitly acknowledged

		analyses, and results from similar studies	
Generalisability	21	Discuss the generalisability (external validity) of the study results	Discussion, p.12, lines 388–391: generalizability limited by single-center design, tertiary referral population, and predominantly idiopathic TN cohort
**OTHER INFORMATION**			
Funding	22	Give the source of funding and the role of the funders for the present study and, if applicable, for the original study on which the present article is based	Funding statement, p.12, line 418: "This research received no external funding."

Notes on STROBE-RECORD Extension (Electronic Health Records)

This study used data routinely collected from a hospital electronic medical record (EMR) system. The following STROBE-RECORD items are addressed:

RECORD Item	Description	Location
1.1	The methods of study design and any electronic health record linkage should be clearly described	Data Collection, p.5, lines 153-1546: data source identified as hospital EMR
6.1	The study setting, including the name and description of the databases used	Materials and Methods, p.3: tertiary pain clinic, Ankara Bilkent City Hospital; data from hospital EMR system
6.2	If the study involved linkage of databases, the methods of linkage and any validation	Not applicable (single-database study)
7.1	A description of the validation studies of the codes or algorithms used to identify cases	Diagnoses established by clinical evaluation per ICHD-3 criteria (Patient Selection, p.3); NRS and BNI recorded prospectively by treating physicians at each follow-up visit
12.1	Authors should describe the extent to which the investigators had access to the database population used to create the study population	Data Collection, p.5: all consecutive eligible procedures within the study period were included; no sampling
13.1	Counts of patients in each stage of the study should reflect the linkage process	Figure 1, p.4: full patient flow from assessed to analysed
19.1	The authors should discuss the implications of using data not collected primarily for the research question	Discuusion, p.12: retrospective design and EMR-based data collection acknowledged as limitations; outcome measures (NRS, BNI) were recorded as part of routine clinical practice
